# A non-synonymous SNP with the allele frequency correlated with the altitude may contribute to the hypoxia adaptation of Tibetan chicken

**DOI:** 10.1371/journal.pone.0172211

**Published:** 2017-02-21

**Authors:** Sichen Li, Diyan Li, Xiaoling Zhao, Yan Wang, Huadong Yin, Lanyun Zhou, Chengling Zhong, Qing Zhu

**Affiliations:** Farm Animal Genetic Resources Exploration and Innovation Key Laboratory of Sichuan Province, Sichuan Agricultural University, Chengdu, Sichuan Province, P. R. China; Sichuan University, CHINA

## Abstract

The hypoxia adaptation to high altitudes is of considerable interest in the biological sciences. As a breed with adaptability to highland environments, the Tibetan chicken (*Gallus gallus domestics*), provides a biological model to search for genetic differences between high and lowland chickens. To address mechanisms of hypoxia adaptability at high altitudes for the Tibetan chicken, we focused on the Endothelial PAS domain protein 1 (*EPAS1*), a key regulatory factor in hypoxia responses. Detected were polymorphisms of *EPAS1* exons in 157 Tibetan chickens from 8 populations and 139 lowland chickens from 7 breeds. We then designed 15 pairs of primers to amplify exon sequences by Sanger sequencing methods. Six single nucleotide polymorphisms (SNPs) were detected, including 2 missense mutations (SNP3 rs316126786 and SNP5 rs740389732) and 4 synonymous mutations (SNP1 rs315040213, SNP4 rs739281102, SNP6 rs739010166, and SNP2 rs14330062). There were negative correlations between altitude and mutant allele frequencies for both SNP6 (rs739010166, r = 0.758, p<0.001) and SNP3 (rs316126786, r = 0.844, *P*<0.001). We also aligned the *EPAS1* protein with ortholog proteins from diverse vertebrates and focused that SNP3 (Y333C) was a conserved site among species. Also, SNP3 (Y333C) occurred in a well-defined protein domain Per-AhR-Arnt-Sim (PAS domain). These results imply that SNP3 (Y333C) is the most likely casual mutation for the high-altitude adaption in Tibetan chicken. These variations of *EPAS1* provide new insights into the gene’s function.

## Introduction

Low oxygen is one of the more severe environmental challenges for animals living in high-altitude regions such as the Tibetan Plateau, where the average altitude is above 4000 m. The partial pressure of oxygen at 4000 m is approximately 50% of that at sea-level, and the hypoxia imposes severe constraints on aerobic metabolism and leading to high-altitude illness[1, 2][3] and the mechanisms of hypoxic adaptation [4].

The Tibetan chicken (*Gallus gallus;* TC) is an aboriginal breed found in the Qinghai-Tibetan Plateau (QTP), where the altitude range from 2200 to 4100 m. They are smaller, mature at older ages and lay smaller eggs than breed at lower altitude (Table A in S1 File). Also, TC has higher red blood cells numbers, blood oxygen affinity, and hemoglobin concentrations [5], and loser mean corpuscular volume. Hatchability at the high altitude is greater for TC [6] than that of lowland chickens [7] and they are is less sensitive to pulmonary hypertension syndrome (PHS) and hypocapnia [8, 9]. It is obviously that TC process unique physiological properties and there provide a model for studying genetic mechanisms of hypoxia adaptation.

Endothelial PAS domain protein 1 (EPAS1) (also known as hypoxia inducible factor-2a (HIF-2a)), belongs to a member of the transcription factor family characterized by a basic helix-loop-helix (bHLH) and by a (Per-AhR-Arnt-Sim) PAS domain. Thus, it plays a prominent role in the process of adaptation to hypoxia and in the promotion of livability during hypoxia stress [10, 11]. *EPAS1* is a prime regulator during chronic hypoxia and directly regulates key genes such as Erythropoietin (*EPO*), and vascular endothelial growth factor (*VEGF*) [12]. Also, *EPAS1* has been implicated processes such as erythropoiesis, iron homeostasis, pulmonary hypertension and vascular permeability [13, 14].

Genome-wide scans and whole genome re-sequencing analyses to identify selection signatures of high-altitude adaptation in vertebrates have been performed across a range of species, including humans[15], dogs [16, 17], grey wolves [18], yaks [19], Tibetan antelopes [20], and cattle [21]. In the humans, dogs, and grey wolf convergent evolution for *EPAS1* gene has been noted [15–18]. Because of an association between the allele frequency of the SNPs near or in *EPAS1* and the altitude. Other studies have focused on the effects of mitochondrial DNA polymorphisms in adaptation of TC to high-altitude. *MT-CO3* [22] and *ATP-6* [23] are candidate genes of hypoxia adaptation. Genes involved in the Ca^2+^ signaling pathway might be related to the adaptation of TC to hypoxic conditions at high altitude[24], and genome re-sequencing, also identified unique hypoxia adaptation of TC, especially genes related to development of the cardiovascular and respiratory systems, DNA repairs, responses to radiation, inflammation, and immune responses [24].

In the present study, we hypothesis that the sequence variations in the *EPAS1* gene may contribute to the high altitude adaptation in TC. Here, we compared on the polymorphisms of *EPAS1* coding sequence in populations that included 157 Tibetan chickens and 139 lowland chickens to determine whether there were genetic differences between high and low altitude chickens in *EPAS1* gene.

## Materials and methods

### Sampling

In total, 157 Tibetan chickens (TC) were collected from 8 different locations in Qinghai, Tibet, Yunnan, and Sichuan provinces. They included 4 Tibet areas (Shigatse (RKZ), Lhoka (SN), Lhasa (LS), and Nyingchi (LZ)), 2 Sichuan areas (Garze (GZ) and Aba (AB)), 1 Yunnan area (Diqing (DQ)), and 1 Qinghai area (Haibei (QH)). Also, 139 lowland chickens (LC) from 7 different Chinese native chicken breeds were sampled (below 1800 m). Details of sample number, breed, altitude, latitude, and longitude are provided in Table 1. A sample of 1 mL blood from each individual was obtained from the brachial vein. No animal was injured in during capture and blood collection. The protocol was approved by the Committee of the Care and Use of Laboratory Animals of the State-level Animal Experimental Teaching Demonstration Center of Sichuan Agricultural University (Approval ID: DKY-S20143136).

**Table 1:** Altitude, longitude and latitude of the sampling locations for the 8 Tibetan and 7 Lowland chicken populations.

Breed	Sampling location	Population	Sample Size	Altitude (m)	Longitude (E)	Latitude (N)
Tibetan						
	Shigatse,Tibet	Shigatse (RKZ)	30	3900	89°60'	28°92'
	Lhoka,Tibet	Lhoka (SN)	14	3700	90°03'	28°27'
	Lhasa,Tibet	Lhasa (LS)	24	3650	91°01'	29°26'
	Garze,Sichuan	Garze (GZ)	16	3390	99°22'	28°34'
	Aba,Sichuan	Aba (AB)	25	3300	102°33'	31°27'
	Diqing,Yunnan	Diqing (DQ)	13	3280	99°53'	28°08'
	Haibei,Qinghai	Haibei (QH)	7	2700	100°86'	36°85'
	Nyingchi,Tibet	Nyingchi (LZ)	20	2400	94°26'	29°25'
Lowland						
Emei black	Emei,Sichuan	Emei (EM)	23	1800	103°41'	29°49'
Shimian	Yaan,Sichuan	Shimian (SM)	14	1120	102°13'	29°40'
Jiuyuan black	Wanyuan,Sichuan	Jiuyuan (JY)	25	900	108°21'	31°84'
Pengxian yellow	Yaan,Sichuan	Pengxian (PX)	12	600	102°98'	29°98'
Blue-shelled Dongxiang	Yaan,Sichuan	Dongxiang(LK)	14	600	102°98'	29°98'
Muchuan black-bone	Muchuan,Sichuan	Muchuan (MC)	20	540	103°90'	29°02'
Wenchang	Wenchang,Hainan	Wenchang(WC)	31	10	110°87'	19°72'

### DNA extraction, PCR amplification, and genotyping

DNA was extracted by the phenol-chloroform method [25]. The high altitude DNA pool was constructed from 50 samples, each containing 100 ng DNA from randomly selected TC. The LC pool was constructed in the same way. These two gene pools were used to detect SNPs.

After blasting chicken *EPAS1* mRNA sequence (Ensembl accession ENSGALT00000016253) in the chicken genome, we designed 15 primer pairs (Table 2) to amplify the 15 exons using Primer V6.0 software [26]. PCR was performed in 25 μL of reaction volume. The ingredient contain 50 ng DNA template, 1×buffer (including 1500 μmol L^-1^ Mg_2_Cl_2_, 200 μmol L^-1^ dNTPs, and 1.5 U of Taq DNA polymerase) and 1 μmol L^-1^ of each primer. Besides, the PCR procedure was as following: initial denaturation for 5 min at 9500, followed by 35 cycles of 95°C for 30 s; annealing at prescribed annealing temperature for 30 s; and primer extension at 72°C for 45 s. The final extension was performed at 72°C for 7 min. PCR products were sequenced on an ABI 3730 DNA sequencer. Then, the SNPs were determined and pairs of 4 primers (P1, P7, P12, and P14) were used for individual genotyping. PCR procedures and systems were the same as above.

**Table 2:** Primer information for detecting SNPs in coding regions.

Names	Sequences (5'-3')	Sizes of product (bp)	Annealing temperatures (°C)	Target region	Purpose
P1	F^1^:CAAGGGGTCTGAAGGCTGAT	534	55	Exon1	Identification of SNP and individual genotyping
	R^2^:TGGGAAAACAAAACAAAAACTG				
P2	F:GTTGCCTTGTTATTGGATGTGAT	473	56	Exon2	Identification of SNP
	R:GGGGGAGTTGTCTTTTCTTGAT				
P3	F:CCCCCACTAATTGTCTCCTG	395	55	Exon3	Identification of SNP
	R:TCCACCTGTTTTAACTTGATGAA				
P4	F:GATGGTCTCTGCAGCTATGTTAC	422	55	Exon4	Identification of SNP
	R:AAGTGTGAGGAGGGCAAGTGT				
P5	F:GAGTCGGTTTTTCGTTCACAA	443	55	Exon5	Identification of SNP
	R:AGCAGGGGTACCATTTTCTCTAA				
P6	F:ACTGTGGTTTTAATCCCTTTTGTT	263	55	Exon6	Identification of SNP
	R:CTACTGGTTGTGGCTTATTATTTG				
P7	F:ATTGGCCACCCTGTTGATG	579	55	Exon7	Identification of SNP and individual genotyping
	R:AGAAGAAAAAGGCTATGGTGTAAT				
P8	F:GGAACTGTGCCTTGCCTGTCA	530	57.8	Exon8	Identification of SNP
	R:GCCTGGTTCTGCCCTCATTCA				
P9	F:AGGGCAGAACCAGGCTTTTT	420	55	Exon9	Identification of SNP
	R:TGGGATTTGCATAGAGAACATAAC				
P10	F:ACTGTAGTTGTCTTGGCTTCTTAT	438	55	Exon10	Identification of SNP
	R:AGGCTGCTGCTTCTATTTGT				
P11	F:AGCTGGCCACTTTCCTTTAC	740	55	Exon11	Identification of SNP
	R:AGCCATGCCTGTCTCCTT				
P12	F:GTGGGATGACTGACAAAAGGAA	397	55	Exon12	Identification of SNP and individual genotyping
	R:TGAGGTGGGTTAATGACAGATGG				
P13	F:GAGTGGGGTAATCATAAGAA	434	55	Exon13	Identification of SNP
	R:ATTTGCCTCCCACATAAG				
P14	F:TGTGCAGGCATTTCAGAG	443	55	Exon14	Identification of SNP and individual genotyping
	R:TGCCAACATTTTTCAGC				
P15	F:GGTATAAATAGAAGAGGGAAGAGA	503	55	Exon15	Identification of SNP
	R:ATAAAGGGAGGGTAATCAAACT				

^1.2^ F and R are the abbreviation of forward and reverse primer, respectively.

### Data analysis

The sequences were edited and aligned by DNAstar Software (DNAstar Inc. Madison, WI, USA). Sequence variations were identified using MEAG5 [27]. The complete *EPAS1* sequence of Red Jungle Fowl (*G*.*g gallus*, Ensemble accession number: ENSGALG00000010005) was used as reference for determining the variable sites of chicken. Hardy-Weinberg equilibrium, pairwise linkage disequilibrium (LD), and association analysis were conducted by Haploview software (version 4.2, http://www.broad.mit.edu/mpg/haploview/). We calculated the nucleotide diversity (Pi) and average number of nucleotide differences (K) for the TC and LC populations by DNAspV5 [28]. The Fixation index (*F*_*st*_) between the high (≥3300m) and low altitude groups (≤600m) were calculated by Arlequin 3.5 [29].

Differences of allele frequencies between TC and LC groups were analyzed using Pearson’ Chi-square test, odds ratio (OR), 95% confidence intervals (95% CLs), and MAF (Minor allele frequencies) were calculated. Correlation coefficients between the altitude and frequencies of mutant alleles were calculated. All these parameters were calculated by SPSS software, Version 22 [30] and *P* < 0.05 was considered as statistical significance.

### Protein secondary structure prediction and variable sites analysis

The protein domains of *EPAS1* were predicted by the Pfam web service [31]. The EPAS1 ortholog proteins of 16 vertebrates were retrieved from Ensembl [32]. Multiple sequence alignments were performed by Clustal X (V2.1) [33] and displayed by Jalview (v2.8) [34]. To find the best model for a Maximum Likelihood (ML) based tree, we use Prottest V3.4 to compare all models and chose the ML tree as starting topology. Then we used BEAST V1.8.3 to generate the trees by the best model. To find the best fit model, we use Prottest V3.4 [35] to exam all the models and chose the Maximum likelihood tree as starting topology. Then BEAST 2 [36] were used to generate the trees by the best model. The protein sequence of chicken *EPAS1* was searched in Ensembl database (ENSGALP00000016234).

## Results

### Sequence variations in *EPAS1* gene

**Fig 1:**
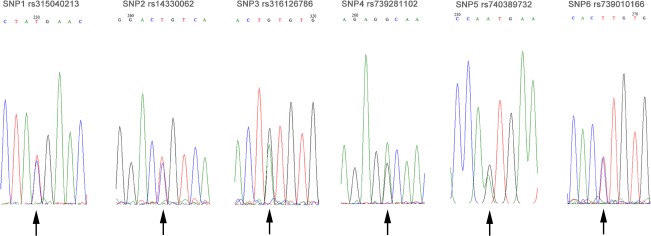
The sequencing images of 6 SNPs in the coding region of chicken *EPAS1* gene. The position and base substitutions in coding regions refer to the sequence of ensemble ENSGALT00000016253.

The length of *EPAS1* exon sequences was 2730 bp (Ensembl accession numbers ENSGALT00000016253; no insertion/deletions were detected). A total of 6 single-nucleotide polymorphisms (SNPs) were found in the exon of *EPAS1* gene from the 15 chicken populations (Table 3 and Fig 1). There were four synonymous mutations (SNP1: rs315040213, SNP2: rs14330062, SNP4: rs739281102, and SNP6: rs739010166) and two non-synonymous substitutions (SNP3: rs316126786 and SNP5: rs740389732).

**Table 3:** Four synonymous mutations and two non-synonymous mutations in *EPAS1*.

SNP locus^1^	Genomic location^2^	Nucleotide mutations		Amino acid mutations	
		Reference allele	Mutation allele	Reference	Mutation
SNP1 rs315040213	Chr3:25981719 (+)	C	T	Tyr	Tyr
SNP2 rs14330062	Chr3:25976150 (+)	T	C	Thr	Thr
SNP3 rs316126786	Chr3:25976203 (+)	A	G	Tyr	Cys
SNP4 rs739281102	Chr3:25980904 (+)	A	G	Arg	Arg
SNP5 rs740389732	Chr3:25981687 (+)	A	G	Met	Val
SNP6 rs739010166	Chr3:25981719 (+)	C	T	Thr	Thr

^1^ SNP loci are exported from dbSNP.

^2^The genomic location of *EPAS1* mutation is based on Ensembl *Gallus gallus* version 84.4 (galgal4) Chromosome 3.

### SNP genotypes

**Fig 2:**
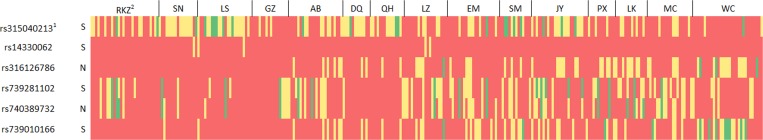
All samples genotypes. These genotypes were derived from Sanger sequencing of 157 Tibetan and 139 Lowland chickens and were marked as red block (homozygous reference, abbreviated as ref), yellow block (heterozygote, abbreviated as het), and green block (homozygote alternative, abbreviated as alt). N: non-synonymous; S: synonymous.^1^The mutation sites of *EPAS1*. ^2^The abbreviations of sample locations, shown are in Table 1.

Genotype frequencies are presented in Table 4 and Fig 2. To detect SNP sites which had putative associations with a high-altitude environment, we focused on genotypes which difference (*P* < 0.05) between TC and LC. Excluding SNP2 (*P* = 0.062), SNP4 (*P* = 0.108, OR = 0.623, 0.408 ≤ 95% CI ≤ 0.952), and SNP5 (*P* = 0.119, OR = 0.675, 0.437 ≤ 95% CI ≤ 1.045), the other SNPs, which were the SNP1 (*P* = 0.001, OR = 1.826, 1.246 ≤ 95% CI ≤ 2.676), SNP3 (*P* = 2.16E-06, OR = 0.257, 0.142 ≤ 95% CI ≤ 0.464), and SNP6 (*P* = 3.76E-07, OR = 0.212, 95% CI = 0.120–0.374) were different between TC and LC groups. Results of odds ratios shared that the probability of the ability to adapt hypoxia for chickens with a C allele at SNP1 polymorphic site was more than 1.826 times that of chickens with T allele. The odds ratios of allele A at SNP3 and allele C at SNP6 were less 0.257 and 0.212 fold than chickens with G and T alleles, respectively.

**Table 4:** Genotype of the *EPAS1* gene SNPs.

SNP locus	Genotypes			*p* value in Pearson Chi-square test
SNP1 rs315040213	CC	CT	TT	0.001
TC^1^	74 (47.10%)^3^	70 (44.50%)	13 (8.20%)	
LC^2^	94 (67.60%)	36 (25.80%)	9 (6.40%)	
SNP2 rs14330062	TT	TC	CC	0.062
TC	152 (96.80%)	5 (3.10%)	0 (0%)	
LC	139 (100%)	0 (0%)	0 (0%)	
SNP3 rs316126786	AA	AG	GG	2.16E-06
TC	142 (90.40%)	14 (8.90%)	1 (0.60%)	
LC	94 (67.60%)	42 (30.20%)	3 (2.10%)	
SNP4 rs739281102	AA	AG	GG	0.108
TC	119 (75.70%)	30 (19.10%)	8 (5%)	
LC	90 (64.70%)	38 (27.30%)	11 (7.90%)	
SNP5 rs740389732	AA	AG	GG	0.119
TC	122 (77.70%)	26 (16.50%)	9 (5.70%)	
LC	94 (67.60%)	36 (25.80%)	9 (6.40%)	
SNP6 rs739010166	CC	CT	TT	3.76E-07
TC	141 (89.80%)	15 (9.50%)	1 (0.60%)	
LC	91 (65.40%)	37 (26.60%)	11 (7.90%)	

^1^TC and

^2^LC abbreviations for Tibetan and lowland chickens, respectively.

^3^Values represent the genotype number and percentage of the population.

### Correlation coefficients between altitudes and allele frequencies of the *EPAS1* gene

Table 5 contains the observed allelic frequencies of each polymorphic site for each sample from the 15 populations (Allele and genotype frequencies of all SNPs were shown in Tables B, C, and D in S1 File.). We found that SNP2 was unique to TC with the allele frequency of 1.5%. The other five SNPs (SNP1, SNP4 and SNP6, SNP3, and SNP5) were shared by TC and LC. Using Pearson chi-square test and Fisher exact test except the SNP5 (*P* = 0.077), the other four SNPs whose allele frequencies were significantly different between TC and LC, implying that these four SNPs were associated with high-altitude adaptation. Notably, The non-synonymous substitutions SNP3 (*P* = 1.94E-06) was significantly different between TC and LC. There were significant negative linear correlations between altitude and the frequency of mutant allele at SNP6 (rs739010166, r = 0.758, p<0.001) and SNP3 (rs316126786, r = 0.844, *P*<0.001). There was no correlation for the other SNPs.

**Table 5:** Allele frequency, correlation and odds ratio of mutation loci in the *EPAS1* gene.

SNP sites	Allele distribution			*p* value in Pearson Chi-square test	OR^1^	95% CI^2^	MAF^3^	*R*	Pearson *r*	*P*-value^8^
	Allele	TC^5^	LC^6^							
SNP1 rs315040213	C	218 (69.4%)^7^	224 (80.5%)	0.001	1.826	1.246-~2.676	0.25337	0.231	0.48	0.07
	T	96 (30.6%)	54 (19.4%)							
SNP2 rs14330062	T	5 (1.5%)	0 (0%)	0.034	NA^4^	NA-NA	0.0084	0.132	0.363	0.184
	C	309 (98.4%)	278 (100%)							
SNP3 rs316126786	A	298 (94.9%)	230 (82.7%)	1.94E-06	0.257	0.142–0.464	0.1081	0.712	0.844	0.000*
	G	16 (5%)	48 (17.2%)							
SNP4 rs739281102	A	268 (85.3%)	218 (78.4%)	0.028	0.623	0.408–0.952	0.179	0.223	0.472	0.076
	G	46 (14.6%)	60 (21.5%)							
SNP5 rs740389732	A	270 (85.9%)	224 (80.5%)	0.077	0.675	0.437–1.045	0.1655	0.17	0.413	0.126
	G	44 (14%)	54 (19.4%)							
SNP6 rs739010166	C	297 (94.5%)	219 (78.7%)	9.53E-09	0.212	0.120–0.374	0.1283	0.575	0.758	0.001*
	T	17 (5.4%)	59 (21.2%)							

^1^OR represents odds ratio

^2^Cl represents confidence interval

^3^MAF represents the minor allele frequency.

^4^ NA means cannot be calculated.

^5^ TC abbreviations for Tibetan chicken.

^6^ LC abbreviations for Lowland chicken.

^7^ Values represent the allele frequency and percentage in the population.

^8^*P*-value was calculated by Pearson r.

*represent significant level *P*<0.05.

**Fig 3:**
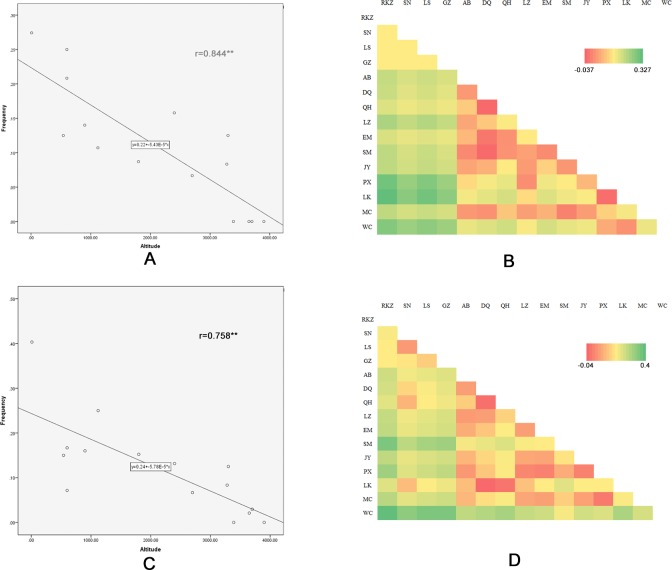
The correlation analysis between frequencies and the altitude for SNP3 and SNP6. (A) Plot of the correlation analysis between frequencies of the SNP3 and the altitudes among fifteen population, “*r*” represents the correlation coefficient (*r* = 0.844, *P*<0.001). (B) The Fst of 15 different populations from TC and LC were calculated based on SNP3 of EPAS1 gene. (C) Plot of the correlation analysis between frequencies of the SNP6 and the altitudes among fifteen chicken population, “*r*” represents the correlation coefficient (*r* = 0.578, *P* = 0.001). (D) The Fst of 15 different populations from TC and LC were calculated based on SNP6 of *EPAS1* gene.

There was a striking change of allele frequency along with the altitude elevation in SNP3. As shown in Fig 3A, the high mutant allele frequencies of SNP3 (Y333C) in the populations WC (27.4%, n = 31, 10 m), LK (25%, n = 14, 600 m), and PX (20.8%, n = 12, 600 m) were found. Then, the frequencies declined in the populations JY (14%, n = 25, 900 m), SM (10.7%, n = 14, 1120 m), and EM (8.7%, n = 23, 1800 m). And they dropped the populations QH (6.7%, n = 15, 2700 m) and DQ (8.3%, n = 12, 3280 m). Moreover, when the altitude was more than 3,400 m, the mutation essentially disappeared, as shown in the populations GZ (0%, n = 16, 3390 m), LS (0%, n = 24, 3650 m), SN (0%, n = 14, 3700 m), and RKZ (0%, n = 30, 3900 m). The same tendency was found in SNP6 (Fig 3C).

These results suggested that the frequencies of SNP3 and SNP6 in *EPAS1* decreased with the elevation of altitude.

### The Hardy-Weinberg equilibrium, nucleotide diversity, and population differentiation of the *EPAS1*

To test whether all of the SNPs at *EPAS1* loci were in equilibrium in each population, the Hardy-Weinberg equilibrium (HWE) tests was performed on each SNP locus (Table 6). Except for SNP4 and SNP5, all alleles in TC were in accordance with Hardy-Weinberg equilibrium (P>0.05). In contrast, there was a departure from Hardy-Weinberg equilibrium (*P* < 0.05) for LC in SNP1, SNP4, SNP5, and SNP6. Notably, SNP3 in both populations was in accordance with Hardy-Weinberg equilibrium. Besides, there were no significant diferences between TC and LC for nucleotide diversity (Pi) and the average number of nucleotide differences (K) (Table E in S1 File). Then we calculated the LD block of Tibetan chicken and Lowland chicken and we found close linkage between SNP3 and SNP6 in TC (Figure A in S1 File). Besides, the observed heterozygosity of all SNPs was from 0.03 to 0.44 in TC and from 0 to 0.30 in LC (Table 6). The minor allele frequencies (MAF) of all mutations were more than 0.005 (Table 5).

**Table 6:** The Hardy-Weinberg equilibrium (HWE) tests of the candidate SNPs in *EPAS1* in TC and the LC.

SNP locus	TC^4^				LC^5^				*F*_*st*_	*P*-value^3^
	*χ*^2^	*Pearson's p*	*Ho*^*1*^	*He*^*2*^	*χ*^2^	Pearson's p	*Ho*	*He*		
SNP1 rs315040213	0.396	0.528	0.44586	0.42588	4.141	0.0418*	0.25899	0.31416	0.18057	<0.000**
SNP2 rs14330062	0.001	0.839	0.03185	0.03144	0	1	0	0	0.01252	0.16031
SNP3 rs316126786	0.955	0.328	0.08917	0.09703	0.461	0.497	0.30216	0.28673	0.22956	<0.000**
SNP4 rs739281102	8.735	0.0031**	0.19108	0.25087	5.143	0.023*	0.27338	0.33971	0.0224	0.07038
SNP5 rs740389732	15.36	0.00090**	0.16561	0.24175	4.141	0.041*	0.25899	0.31416	0.02805	0.02933
SNP6 rs739010166	0.707	0.400	0.09554	0.10275	5.78	0.016*	0.26619	0.33558	0.27826	<0.000**

*Represent significant *P*-value of less than 0.05.

** represent significant *P*-value of less than 0.001.

^1.2^ Ho and He represent the observed heterozygosity and expected heterozygosity.

^3^ The P-value is calculated by *Fst* value.

^4^ TC abbreviations for Tibetan chickens.

^5^ LC abbreviations for Lowland chickens.

To fully understand the population differentiation that occurred between TC and LC, we analyzed the distribution of alleles at the *EPAS1* for the high altitude (≥3300m) and low altitude groups (≤600m). The Fixation index (*F*_*st*_) was calculated to evaluate the population differentiation degree. As shown in Table 6, the *F*_*st*_ values for SNP1 (0.181, *P* < 0.001), SNP3 (0.229, *P* < 0.001), and SNP6 (0.278, *P* < 0.001) for two groups reflected remarkable population differentiation.

### Protein secondary structure prediction and non-synonymous sites analysis

**Fig 4:**
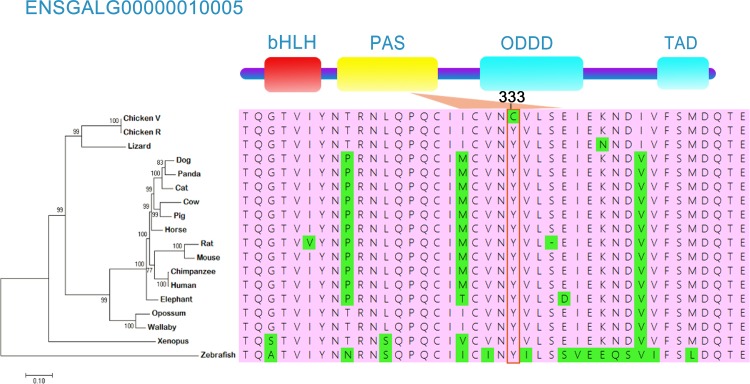
*EPAS1* mutation in coding regions. Structural and evolutionary analysis of the 4 amino acid variants. The protein coordinate is based on Ensembl ID ENSGALP00000016234. The upper layer displayed the Pfam domains of the protein. The orthologous proteins from 17 different vertebrates were aligned with the residues shown as green. Cow, ENSBTAP00000004836; pig, ENSSSCP00000009011; human, ENSP00000406137; dog, ENSCAFP00000003819; elephant, ENSLAFP00000010336; mouse, ENSMUSP00000024954; opossum, ENSMODP00000001136; lizard, ENSACAP00000004025; xenopus, ENSXETP00000031612; zebrafish, ENSDARP00000074832; wallaby, ENSMEUT00000004049; horse, ENSECAT00000015683; cat, ENSFCAT00000013633; rat, ENSRNOT00000034991; panda, ENSAMET00000006660; chimpanzee, ENSPTRT00000022124. The ML tree is shown in the left area. (PAS) Per-Arnt-Sim; (HIF) hypoxia-inducible factor; (ODDD) O_2_-dependent degradation domain; (TAD) C-terminal transactivation domain.

SNP3 (Y333C) was located in a well-defined protein domain (PAS domain, Fig 4). Using the crystal structure model of PAS domain for human *EPAS1*, we found that mutation SNP3 in chicken (Y333C) occurred in a beta sheet which may affect the thermodynamic stability of the domain. To evaluate the its functional, we aligned the mutant EPAS1 protein with its ortholog proteins of diverse vertebrates (Fig 4) and found that the amino acid mutation (Y333C) were conserved among species. By contrast, M765V was not found in other species. Because mutant functional effects showed that Y333C was deleterious, while M765V were tolerated implied that Y333C was the most likely casual mutation for the *EPAS1* in LC but not TC.

## Discussion

Long-term exposure to hypoxia reduced metabolic activity, retarded development, and increased embryonic mortality in animal fetes [37] and chronic hypoxia decreased growth of developing chick embryos [38, 39]. The Tibetan chicken, a Chinese native chicken breed, adapts itself well to the low pressure and high altitude plateau environment [7, 40]. Here, we investigated specific SNPs in *EPAS1* gene and discuss their possible contributions to the high-altitude adaptation of the Tibetan chicken.

Amino acid replacement at key sites can difference protein function [41]. *EPAS1* protein has an important role in regulating transcriptional responses to hypoxia in physiological and pathologic conditions including up-regulating the expression of erythropoietin (*EPO*) [42], Thus, it is not surprising that Mutations at *EPAS1* are associated with hematologic phenotypes [43].

Here, we found 4 synonymous mutations and 2 non-synonymous substitutions which resulted in amino acid mutations of Tyr to Cys at SNP3 and Met to Val at SNP5, respectively. Except for SNP5, the other 5 SNPs were significantly different between TC and LC for genotypic frequencies. Both genotypic and allelic results for SNP1, SNP3, and SNP6 revealed significant differences between two chicken groups. Except for SNP4 and SNP5, all the alleles in the TC were in accordance with Hardy-Weinberg equilibrium. While for LC there was a significant departure from Hardy-Weinberg equilibrium (*P* < 0.05) in SNP1, SNP4, SNP5, and SNP6. These results implied an association of SNP1 and SNP6 might be correlate with high-altitude adaptation. Also there is linkage between SNP3 and SNP6 in TC (Figure A in S1 File). The *Fst* values for SNP1, SNP3, and SNP6 of *EPAS1* between the high altitude group (HIC≥3300m) and low altitude group (LOC ≤ 600m) reflected remarkable population differentiation. Overall, both the *Fst* value and the LD block demonstrate a remarkable population differentiation between the TC and LC. We deduce reasons why the previous study[24] did not find SNP3 are that compared to other SNPs, SNP3 may have low frequency in the TC population and sample size, only 40 Tibetan chickens from 3 different areas were involved.

A correlation coefficient is a number that quantifies some type of association between random variables and observed data [44]. Pearson correlation between altitude and allele frequency showed that in TC there was a significant relationship in synonymous mutation SNP6 and non-synonymous mutation SNP3. The frequencies of SNP3 (Y333C) and SNP6 were high in LC but decreased along with the elevation of altitude. The same relationship also found for the *EPAS1* gene in cattle [45]. There was an association of double variants in the oxygen degradation domain (ODDD) of *EPAS1* in Angus cattle with High-altitude pulmonary hypertension (HAPH). These mutations were prevalent in lowland cattle, but they disappeared for the animals located at high altitudes[45]. Like Angus cattle, the mutation we found was located in a well-defined domain and quite conserved.

Human EPAS1 is a basic helix-loop-helix transcription factor that contains a Per-AhR-Arnt-Sim (PAS) domain and shares 48% homology [46, 47]. The transcriptional response to hypoxia appears in several physiological and pathologic conditions, including up-regulation in the expression of erythropoietin (*EPO*) [48]. Mutations at *EPAS1* in humans are associated with hematologic phenotypes [43]. HIF proteins are constantly degraded with sufficient oxygen availability, but one released under hypoxia condition. HIFs are protected by inhibition of oxygen-dependent hydroxylation of specific residues in the oxygen-dependent degradation domain under hypoxic condition. This prevents interactions with the von Hippel-Lindau ubiquitin ligase complex and proteasome destruction. *HIF2a* is found in all human tissues and its effects on downstream cascades include regulation of angioenic factors *VEGF* and *TGFa*, and cell permeability and stimulation of erythropoietic and glycolytic protein [46, 49, 50]. Clinical studies found that several gain-of-function mutations that occurred in the primary hydroxylation site (Pro531) at *EPAS1* caused erythroctosis and pulmonary hypertension [51].While, the unique *EPAS1* mutation in the Tibetan people were associated with lower hemoglobin concentrations [52, 53], suggesting that it played a loss-of-function role to high blood viscosity and cardiovascular disorders. As erythrocytosis is a common symptom of chronic mountain sickness which can lead to high blood viscosity and cardiovascular disorders, a decrease of hemoglobin level may provide a protective mechanism for Tibetan people [43]. Nonetheless, it is worth noting that all *EPAS1* variations detected preciously in the Tibetan population are in introns [52, 53].

Transgenic mice carrying a G536W variant in *EPAS1* developed high right ventricular systolic pressure and medial hypertrophy of pulmonary arteries in addition to erythrocytosis under normoxic condition [54]. The key amino acid mutation G305S was identified at *EAPS1* PAS domain in dogs was predicted to be damaging, which is also likely to cause the loss of function of *EPAS1* and it is also associated with blood flow resistance [16]. Recently, the missense mutation Q597L was identified in *EPAS1* in the Tibetan Cashmere goat, which occurred next to the hypoxia-inducible factor-1 (*HIF-1*) domain, and was exclusively enriched in the high-altitude population [55]. Thus, *HIF2a* dysregulation is well recognized in association with pulmonary hypertension, frequency in context of erythrocytosis, with or without severe hypoxemia. Here, we found a non-synonymous mutation SNP3 (Y333C) in PAS domain which is similar to the mutation in dogs and Angus cattle. In order to adapt to the same selection pressure, mutations that produce an adaptive change in one species may preclude possibilities in other species because of differences in genetic background and experiments involving resurrected ancestral proteins have revealed continuous effects of historical substitutions [56]. Although the function of mutation (SNP3 Y333C) in chickens is unknown, based on the previously studies, we inferred that SNP3 (Y333C) might also be harmful to chickens at high altitudes. The lower oxygen and extreme environment selectively swept out mutation SNP3 (Y333C). Taking together, these results indicate that *EAPS1* gene may play a key role in the adaptation of the Tibetan chicken to a high-altitude environment at the molecular level. The adaptation patterns however, may differ in human, dog, grey wolf, and Tibetan Cashmere goat.

In conclusion, genetic analysis in the present study for *EPAS1* gene in TC and LC populations indicated that the non-synonymous SNP3 (Y333C) may have critical role in the high-altitude adaptation of TC. Although the adaptation patterns may not be the same in humans and dogs, these novel variations of *EPAS1* could put new insights into the gene function.

## Supporting information

S1 File The LD Block, production performance, allele frequency and nucleotide diversities. Figure A. The LD block of Tibetan chicken. TC means Tibetan chicken.
Table A. The partial production performance of Tibetan and 7 lowland chicken breeds. The production performance data of 8 breeds of chicken is based on poultry genetic resources in China. Table B. Allele and genotype frequencies of the SNP1 rs315040213 and SNP2 rs14330062 in the EPAS1. “C” represents the reference allele and “T” represents the mutant allele. Numbers represent allele/genotype frequency, with the figures in brackets representing the number of individuals with each genotype. Table C. Allele and genotype frequencies of the SNP3 rs316126786 and SNP4 rs739281102 in the EPAS1. “A” represents the reference allele and “G” represents the mutant allele. Numbers represent allele/genotype frequency, with the figures in brackets representing the number of individuals with each genotype. Table D. Allele and genotype frequencies of the SNP5 rs740389732 and SNP6 rs739010166 in the EPAS1. “A” represents the reference allele and “G” represents the mutant allele. Numbers represent allele/genotype frequency, with the figures in brackets representing the number of individuals with each genotype. “C” represents the reference allele and “T” represents the mutant allele. Numbers represent allele/genotype frequency, with the figures in brackets representing the number of individuals with each genotype. Table E. Nucleotide diversities of EPAS1 gene in Tibetan chicken and Lowland chicken. ^1^Pi is the abbreviation of nucleotide diversity. ^2^K is the abbreviation of average number of nucleotide differences.^3^TC means Tibetan chicken.^4^LC means Lowland chicken.
(DOCX)
