# Supplementary material for: A non-synonymous SNP with the allele frequency correlated with the altitude may contribute to the hypoxia adaptation of Tibetan chicken
Source: PLoS One. 2017 Feb 21;12(2):e0172211. doi: 10.1371/journal.pone.0172211 (PMC5319789; doi:10.1371/journal.pone.0172211)
Supplement: S1 File — Figure A. The LD block of Tibetan chicken. TC means Tibetan chicken. Table A. The partial production performance of Tibetan and 7 lowland chicken breeds. The production performance data of 8 breeds of chicken is based on poultry genetic resources in China. Table B. Allele and genotype frequencies of the SNP1 rs315040213 and SNP2 rs14330062 in the EPAS1. “C” represents the reference allele and “T” represents the mutant allele. Numbers represent allele/genotype frequency, with the figures in brackets representing the number of individuals with each genotype. Table C. Allele and genotype frequencies of the SNP3 rs316126786 and SNP4 rs739281102 in the EPAS1. “A” represents the reference allele and “G” represents the mutant allele. Numbers represent allele/genotype frequency, with the figures in brackets representing the number of individuals with each genotype. Table D. Allele and genotype frequencies of the SNP5 rs740389732 and SNP6 rs739010166 in the EPAS1. “A” represents the reference allele and “G” represents the mutant allele. Numbers represent allele/genotype frequency, with the figures in brackets representing the number of individuals with each genotype. “C” represents the reference allele and “T” represents the mutant allele. Numbers represent allele/genotype frequency, with the figures in brackets representing the number of individuals with each genotype. Table E. Nucleotide diversities of EPAS1 gene in Tibetan chicken and Lowland chicken. 1Pi is the abbreviation of nucleotide diversity. 2K is the abbreviation of average number of nucleotide differences.3TC means Tibetan chicken.4LC means Lowland chicken. (DOCX) [file pone.0172211.s001.docx]

**Table A. The partial production performance of Tibetan and 7 lowland chicken breeds.**

| Breed | Body weight (g) | | Eviscerated yield (%) | | Age at first egg (d) | Egg weight (g) |
| --- | --- | --- | --- | --- | --- | --- |
|  | male | female | male | female |  |  |
| Tibetan | 1585 | 860 | 73.4 | 67.3 | 270 | 42 |
| Emei | 2622 | 1904 | 79.65 | 70.9 | 186 | 54 |
| Shimian | 3650 | 3060 | 69.5 | 65.2 | 216 | 55 |
| Jiuyuan | 2616 | 1763 | 79.7 | 67 | 181 | 55 |
| Pengxian | 3950 | 1880 | 79.1 | 72.12 | 216 | 54 |
| Blue-shelled | 1650 | 1300 | 64.5 | 71.25 | 152 | 50 |
| Muchuan black-bone | 2680 | 2290 | 79 | 69 | 225 | 54 |
| Wenchang | 1800 | 1500 | 75 | 70.3 | 145 | 49 |

The production performance data of 8 breeds of chicken is based on poultry genetic resources in China[[1](#_ENREF_1)] .

**Table B. Allele and genotype frequencies of the SNP1 rs315040213 and SNP2 rs14330062 in the *EPAS1.***

| Breed | SNP1 rs315040213 | | | | | SNP2 rs14330062 | | | | |
| --- | --- | --- | --- | --- | --- | --- | --- | --- | --- | --- |
|  | Genotype | | | Allele | | Genotype | | | Allele | |
|  | C C | C T | T T | C | T | C C | C T | T T | C | T |
| RKZ(30) | 16(0.53) | 12(0.4) | 2(0.07) | 44(0.73) | 16(0.27) | 30(1) | 0(0) | 0(0) | 60(1) | 0(0) |
| SN(17) | 4(0.24) | 12(0.71) | 1(0.06) | 20(0.59) | 14(0.41) | 16(0.94) | 1(0.06) | 0(0) | 33(0.97) | 1(0.03) |
| LS(24) | 5(0.21) | 13(0.54) | 6(0.25) | 23(0.48) | 25(0.52) | 22(0.92) | 2(0.08) | 0(0) | 46(0.96) | 2(0.04) |
| GZ(16) | 13(0.81) | 3(0.19) | 0(0) | 29(0.91) | 3(0.09) | 16(1) | 0(0) | 0(0) | 32(1) | 0(0) |
| AB(24) | 14(0.58) | 9(0.38) | 1(0.04) | 37(0.77) | 11(0.23) | 24(1) | 0(0) | 0(0) | 48(1) | 0(0) |
| DQ(12) | 3(0.25) | 8(0.67) | 1(0.08) | 14(0.58) | 10(0.42) | 12(1) | 0(0) | 0(0) | 24(1) | 0(0) |
| QH(15) | 5(0.33) | 8(0.53) | 2(0.13) | 18(0.6) | 12(0.4) | 15(1) | 0(0) | 0(0) | 30(1) | 0(0) |
| LZ(19) | 14(0.74) | 5(0.26) | 0(0) | 33(0.87) | 5(0.13) | 17(0.89) | 2(0.11) | 0(0) | 36(0.95) | 2(0.05) |
| EM(23) | 16(0.7) | 6(0.26) | 1(0.04) | 38(0.83) | 8(0.17) | 23(1) | 0(0) | 0(0) | 46(1) | 0(0) |
| SM(14) | 7(0.5) | 3(0.21) | 4(0.29) | 17(0.61) | 11(0.39) | 14(1) | 0(0) | 0(0) | 28(1) | 0(0) |
| JY(25) | 10(0.4) | 12(0.48) | 3(0.12) | 32(0.64) | 18(0.36) | 25(1) | 0(0) | 0(0) | 50(1) | 0(0) |
| PX(12) | 9(0.75) | 3(0.25) | 0(0) | 21(0.88) | 3(0.13) | 12(1) | 0(0) | 0(0) | 24(1) | 0(0) |
| LK(14) | 9(0.64) | 5(0.36) | 0(0) | 23(0.82) | 5(0.18) | 14(1) | 0(0) | 0(0) | 28(1) | 0(0) |
| MC(20) | 15(0.75) | 5(0.25) | 0(0) | 35(0.875) | 5(0.125) | 20(1) | 0(0) | 0(0) | 40(1) | 0(0) |
| WC(31) | 28(0.9) | 2(0.06) | 1(0.03) | 58(0.94) | 4(0.06) | 31(1) | 0(0) | 0(0) | 62(1) | 0(0) |

“C” represents the reference allele and “T” represents the mutant allele. Numbers represent allele/genotype frequency, with the figures in brackets representing the number of individuals with each genotype.

**Table C. Allele and genotype frequencies of the SNP3 rs316126786 and SNP4 rs739281102 in the *EPAS1*.**

| Breed | SNP3 rs316126786 | | | | | SNP4 rs739281102 | | | | |
| --- | --- | --- | --- | --- | --- | --- | --- | --- | --- | --- |
|  | Genotype | | | Allele | | Genotype | | | Allele | |
|  | A A | A G | G G | A | G | A A | A G | G G | A | G |
| RKZ(30) | 30(1) | 0(0) | 0(0) | 60(1) | 0(0) | 22(0.73) | 6(0.2) | 2(0.07) | 50(0.83) | 10(0.17) |
| SN(17) | 17(1) | 0(0) | 0(0) | 34(1) | 0(0) | 15(0.88) | 1(0.06) | 1(0.06) | 31(0.91) | 3(0.09) |
| LS(24) | 24(1) | 0(0) | 0(0) | 48(1) | 0(0) | 21(0.88) | 2(0.08) | 1(0.04) | 44(0.92) | 4(0.08) |
| GZ(16) | 16(1) | 0(0) | 0(0) | 32(1) | 0(0) | 12(0.75) | 3(0.19) | 1(0.06) | 27(0.84) | 5(0.16) |
| AB(24) | 18(0.75) | 6(0.25) | 0(0) | 42(0.88) | 6(0.13) | 14(0.58) | 8(0.33) | 2(0.08) | 36(0.75) | 12(0.25) |
| DQ(12) | 10(0.83) | 2(0.17) | 0(0) | 22(0.92) | 2(0.08) | 11(0.92) | 1(0.08) | 0(0) | 23(0.96) | 1(0.04) |
| QH(15) | 13(0.87) | 2(0.13) | 0(0) | 28(0.93) | 2(0.07) | 14(0.93) | 1(0.07) | 0(0) | 29(0.97) | 1(0.03) |
| LZ(19) | 14(0.74) | 4(0.21) | 1(0.05) | 32(0.84) | 6(0.16) | 10(0.53) | 8(0.42) | 1(0.05) | 28(0.74) | 10(0.26) |
| EM(23) | 19(0.83) | 4(0.17) | 0(0) | 42(0.91) | 4(0.09) | 14(0.61) | 7(0.3) | 2(0.09) | 35(0.76) | 11(0.24) |
| SM(14) | 11(0.79) | 3(0.21) | 0(0) | 25(0.89) | 3(0.11) | 9(0.64) | 5(0.36) | 0(0) | 23(0.82) | 5(0.18) |
| JY(25) | 18(0.72) | 7(0.28) | 0(0) | 43(0.86) | 7(0.14) | 16(0.64) | 4(0.16) | 5(0.2) | 36(0.72) | 14(0.28) |
| PX(12) | 7(0.58) | 5(0.42) | 0(0) | 19(0.79) | 5(0.21) | 8(0.67) | 2(0.17) | 2(0.17) | 18(0.75) | 6(0.25) |
| LK(14) | 7(0.5) | 7(0.5) | 0(0) | 21(0.75) | 7(0.25) | 8(0.57) | 5(0.36) | 1(0.07) | 21(0.75) | 7(0.25) |
| MC(20) | 15(0.75) | 5(0.25) | 0(0) | 35(0.875) | 5(0.125) | 10(0.5) | 9(0.45) | 1(0.05) | 29(0.725) | 11(0.275) |
| WC(31) | 17(0.55) | 11(0.35) | 3(0.1) | 45(0.73) | 17(0.27) | 25(0.81) | 6(0.19) | 0(0) | 56(0.9) | 6(0.1) |

“A” represents the reference allele and “G” represents the mutant allele. Numbers represent allele/genotype frequency, with the figures in brackets representing the number of individuals with each genotype.

**Table D. Allele and genotype frequencies of the SNP5 rs740389732 and SNP6 rs739010166 in the *EPAS1*.**

| Breed | SNP5 rs740389732 | | | | | SNP6 rs739010166 | | | | |
| --- | --- | --- | --- | --- | --- | --- | --- | --- | --- | --- |
|  | Genotype | | | Allele | | Genotype | | | Allele | |
|  | A A | A G | G G | A | G | C C | C T | T T | C | T |
| RKZ(30) | 23(0.77) | 4(0.13) | 3(0.1) | 50(0.83) | 10(0.17) | 30(1) | 0(0) | 0(0) | 60(1) | 0(0) |
| SN(17) | 14(0.82) | 2(0.12) | 1(0.06) | 30(0.88) | 4(0.12) | 16(0.94) | 1(0.06) | 0(0) | 33(0.97) | 1(0.03) |
| LS(24) | 22(0.92) | 1(0.04) | 1(0.04) | 45(0.94) | 3(0.06) | 23(0.96) | 1(0.04) | 0(0) | 47(0.98) | 1(0.02) |
| GZ(16) | 12(0.75) | 3(0.19) | 1(0.06) | 27(0.84) | 5(0.16) | 16(1) | 0(0) | 0(0) | 32(1) | 0(0) |
| AB(24) | 15(0.63) | 7(0.29) | 2(0.08) | 37(0.77) | 11(0.23) | 18(0.75) | 6(0.25) | 0(0) | 42(0.88) | 6(0.13) |
| DQ(12) | 11(0.92) | 1(0.08) | 0(0) | 23(0.96) | 1(0.04) | 10(0.83) | 2(0.17) | 0(0) | 22(0.92) | 2(0.08) |
| QH(15) | 14(0.93) | 1(0.07) | 0(0) | 29(0.97) | 1(0.03) | 13(0.87) | 2(0.13) | 0(0) | 28(0.93) | 2(0.07) |
| LZ(19) | 11(0.58) | 7(0.37) | 1(0.05) | 29(0.76) | 9(0.24) | 15(0.79) | 3(0.16) | 1(0.05) | 33(0.87) | 5(0.13) |
| EM(23) | 14(0.61) | 7(0.3) | 2(0.09) | 35(0.76) | 11(0.24) | 16(0.7) | 7(0.3) | 0(0) | 39(0.85) | 7(0.15) |
| SM(14) | 9(0.64) | 5(0.36) | 0(0) | 23(0.82) | 5(0.18) | 8(0.57) | 5(0.36) | 1(0.07) | 21(0.75) | 7(0.25) |
| JY(25) | 17(0.68) | 7(0.28) | 1(0.04) | 41(0.82) | 9(0.18) | 17(0.68) | 8(0.32) | 0(0) | 42(0.84) | 8(0.16) |
| PX(12) | 9(0.75) | 1(0.08) | 2(0.17) | 19(0.79) | 5(0.21) | 9(0.75) | 2(0.17) | 1(0.08) | 20(0.83) | 4(0.17) |
| LK(14) | 9(0.64) | 2(0.14) | 3(0.21) | 20(0.71) | 8(0.29) | 12(0.86) | 2(0.14) | 0(0) | 26(0.93) | 2(0.07) |
| MC(20) | 10(0.5) | 9(0.45) | 1(0.05) | 29(0.725) | 11(0.275) | 15(0.75) | 4(0.2) | 1(0.05) | 34(0.85) | 6(0.15) |
| WC(31) | 26(0.84) | 5(0.16) | 0(0) | 57(0.92) | 5(0.08) | 14(0.45) | 9(0.29) | 8(0.26) | 37(0.6) | 25(0.4) |

“A” represents the reference allele and “G” represents the mutant allele. Numbers represent allele/genotype frequency, with the figures in brackets representing the number of individuals with each genotype.

“C” represents the reference allele and “T” represents the mutant allele. Numbers represent allele/genotype frequency, with the figures in brackets representing the number of individuals with each genotype.

**Table E. Nucleotide diversities of EPAS1 gene in Tibetan chicken and Lowland chicken.**

| Population | Sample size | SNP sites | Pi^1^ | K^2^ |
| --- | --- | --- | --- | --- |
| TC^3^ | 157 | 6 | 0.00188 | 1.150 |
| LC^4^ | 139 | 5 | 0.00295 | 1.590 |
| P-value | NA | 0.058 | 0.139 | 0.101 |

^1^ Pi is the abbreviation of nucleotide diversity.

^2^ K is the abbreviation of average number of nucleotide differences.

^3^ TC means Tibetan chicken.

^4^ LC means Lowland chicken.

**Figure A. The LD block of Tibetan chicken.**


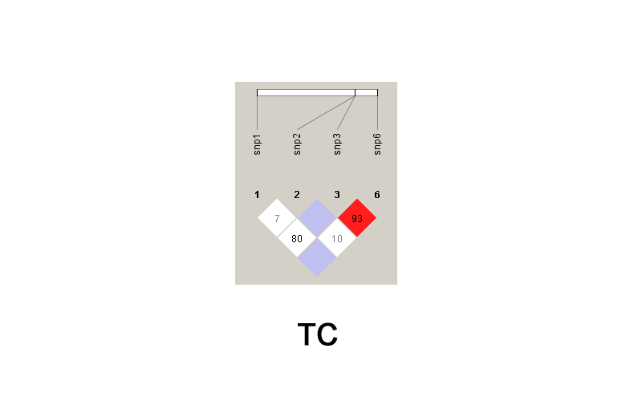


TC means Tibetan chicken.

Genbank Sequence access number.

Gene pool sequence number: grp 5894795.

Gallus_gallus_endothelial_PAS_domain_protein.sqn: KY572133

Individual sequence number: grp 5894798.

Gallus_gallus_endothelial_PAS_domain_protein_1_gene_partial_CDS_exon1.sqn: KY570949 - KY571244

Gallus_gallus_endothelial_PAS_domain_protein_1_gene_partial_CDS_exon7.sqn: KY571245 - KY571540

Gallus_gallus_endothelial_PAS_domain_protein_1_gene_partial_CDS_exon12.sqn: KY571541 - KY571836

Gallus_gallus_endothelial_PAS_domain_protein_1_gene_partial_CDS_exon14.sqn: KY571837 - KY572132

**Reference**

1. Chen G-H, Wang K-H, Wang J-Y, Ding C, Yang N, Dai G, et al. Poultry genetic resources in China. Shanghai Scientific and Technological Press, Shanghai, China. 2004.
